# Supplementary material for: What does it take to learn the rules of RNA base pairing? A lot less than you may think
Source: bioRxiv. 2025 Aug 2:2025.07.31.668042. Preprint. [Version 1] doi: 10.1101/2025.07.31.668042 (PMC12324431; doi:10.1101/2025.07.31.668042)
Supplement: Supplement 1 [file media-1.gz › supplemental_material/src/rscape_v2.6.0.tar.gz!/rscape_v2.6.0/lib/infernal/Manuscripts/filter_thresholds/figs/roc.pdf]

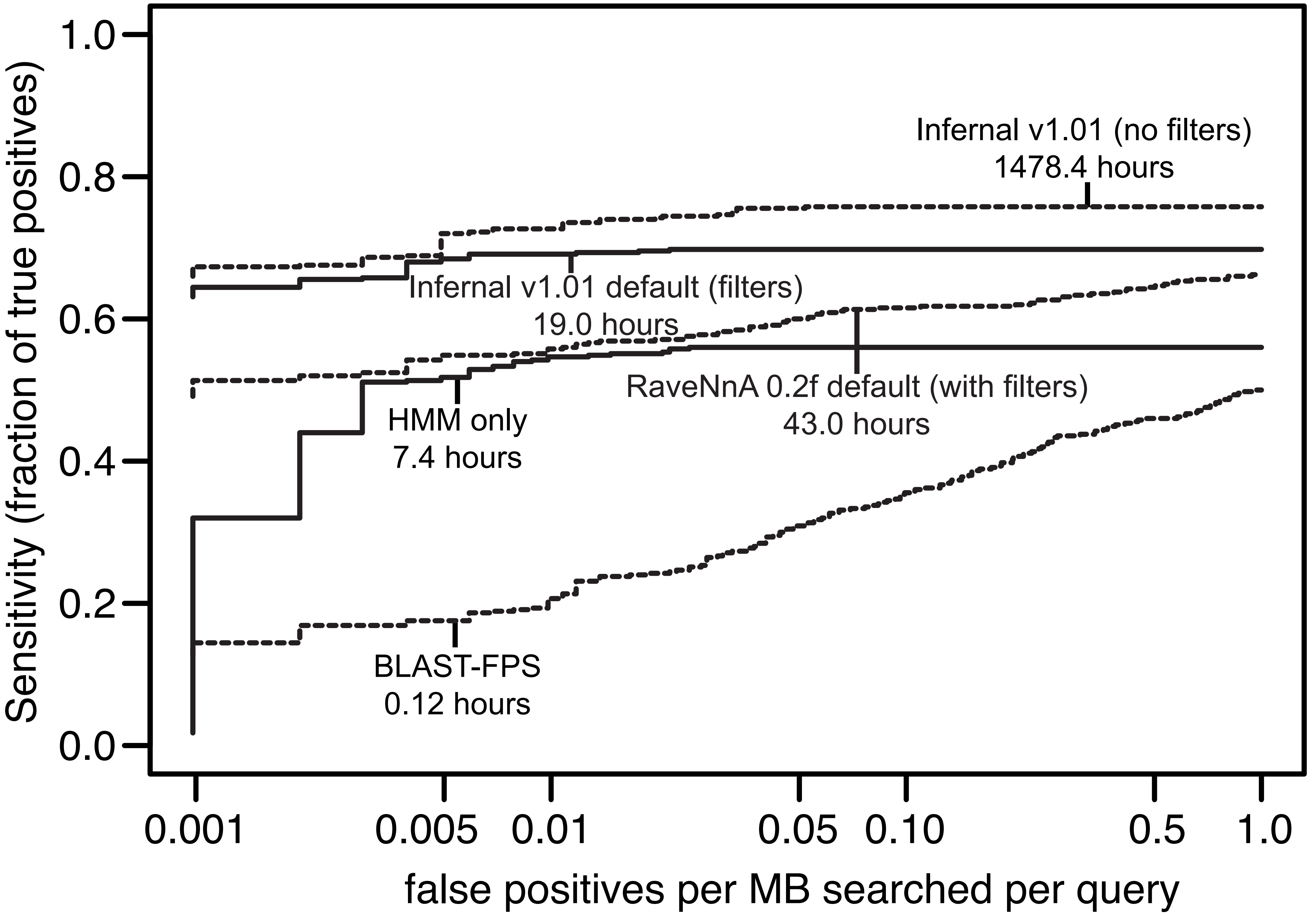

Sensitivity (fraction of true positives)
1.0
0.8
0.6
0.4
0.2
0.0
Infernal v1.01 (no filters)
1478.4 hours
Infernal v1.01 default (filters)
19.0 hours
RaveNnA 0.2f default (with filters)
43.0 hours
HMM only
7.4 hours
BLAST-FPS
0.12 hours
0.001
0.005
0.01
0.05
0.10
0.5
1.0
false positives per MB searched per query
